# The Profile of Selected Antioxidants in Two Courgette Varieties from Organic and Conventional Production

**DOI:** 10.3390/antiox9050404

**Published:** 2020-05-09

**Authors:** Klaudia Kopczyńska, Renata Kazimierczak, Dominika Średnicka-Tober, Marcin Barański, Zdzisław Wyszyński, Katarzyna Kucińska, Aneta Perzanowska, Paweł Szacki, Ewa Rembiałkowska, Ewelina Hallmann

**Affiliations:** 1 Department of Functional and Organic Food, Institute of Human Nutrition Sciences, Warsaw University of Life Sciences, Nowoursynowska 159c, 02-776 Warsaw, Poland; klaudia_kopczynska@sggw.edu.pl (K.K.); renata_kazimierczak@sggw.edu.pl (R.K.); marcin_baranski@sggw.edu.pl (M.B.); ewa_rembialkowska@sggw.edu.pl (E.R.); ewelina_hallmann@sggw.edu.pl (E.H.); 2 Department of Agronomy, Institute of Agriculture, Warsaw University of Life Sciences, Nowoursynowska 159, 02-787 Warsaw, Poland; zdzislaw_wyszynski@sggw.edu.pl (Z.W.); katarzyna_kucinska@sggw.edu.pl (K.K.); aneta_perzanowska@sggw.edu.pl (A.P.); pawel_szacki@sggw.edu.pl (P.S.)

**Keywords:** antioxidants, phenolics, carotenoids, vitamin C, *Cucurbitaceae*, *Cucurbita pepo*, zucchini, courgette, organic production, conventional production

## Abstract

Courgette is considered as a low-calorie vegetable with health-promoting properties. However, scientific publications focused on the profile and content of bioactive compounds in courgette, as well as the potential fruit quality modulating factors, are rare. Due to the high adaptability of courgette to weather and agronomic conditions, it is produced on a global scale. The aim of this study was to analyse the impact of organic versus conventional agronomic practices on the concentration of selected antioxidants in courgette fruits. Fruits of two courgette varieties (Astra Polka and Nimba) produced in an organic and conventional system were tested by high performance liquid chromatography (HPLC) to determine the content of polyphenols (flavonoids and phenolic acids), carotenoids, chlorophylls, and vitamin C. Organic courgette fruits were characterised by their significantly higher content of phenolic acids and flavonoids when compared to the conventionally grown fruit. The organic cultivation might be a good method to increase concentration of bioactive compounds with antioxidant properties in courgette fruits. Nevertheless, the identified trends should be further confirmed, with attention paid to other courgette varieties, as well as to the potential interactions between the plant genotype, agronomic system and the location-specific growing conditions.

## 1. Introduction

*Cucurbitaceae* is a large family of over 800 species, including such species as *Cucumis sativus*, *Cucurbita maxima*, *Cucurbita moschata*, and *Cucurbita pepo.* Courgette, classified as a summer squash, belongs to *Cucurbita pepo* [1,2]. While squashes generally originated from the Americas, this particular variety was developed for the first time in the early 1800s in the North Italy [1,3]. Today, courgette is called summer squash, marrow, or zucchini, depending on the place of its cultivation. Courgette plants produce fruits with the best psychical properties (size, length, weight) for a food technology sector [3]. Thus, the courgette fruits gained significant importance not only on the fresh food market but also as a raw material for various kinds of vegetable-based processed food items [1,2,3,4,5,6,7,8], especially in Mediterranean and European countries [9].

While some species of the *Cucurbitaceae* family (i.e., pumpkin) have been already precisely characterised [10,11,12,13,14], the nutritional value and health-promoting properties of the others (i.e., courgette) are still insufficiently recognised [15]. It is known that courgette fruit contains approximately 93.5–95% of water [16,17] and is thus characterised by very low energy value (21–22 kcal in 100 g f.w.) [16,18], contributed mainly by carbohydrates (2.3–4.2 g/100 g f.w., including 1.3–3.2 g/100 g f.w. of sugars) [16,17,18,19] and proteins (1–2.5 g/100 g f.w.) [16,17,18]. The fibre content in courgette reaches 1.1 g/100 g f.w. [18]. The main minerals in courgette are potassium, magnesium, phosphorus, and calcium, found in higher concentrations in the skin than in the flesh of the fruit [2,17].

The *Cucurbitaceae* are generally considered a moderate source of bioactive compounds with antioxidant properties, but it strongly depends on the species, variety, ripening stage, and other conditions [2]. Some studies have demonstrated antioxidant, anti-inflammatory, neuroprotective, and anticancer properties of vegetables of the *Cucurbitaceae* family [20], which could be linked to the presence of antioxidants, such as phenolics, carotenoids, chlorophylls, and vitamin C, in their composition [21].

Organic production of vegetables is based on natural fertilizers (manure, compost), non-chemical plant protection methods (e.g., pheromone traps, glue boards) and diverse crop rotations. Organic agriculture is defined as a farming method which should contribute to making food systems more sustainable by “combining best environmental and climate action practices, a high level of biodiversity, the preservation of natural resources and the application of high animal welfare standards and high production standards in line with the demand of a growing number of consumers for products produced using natural substances and processes”, as stated in the European Council Regulation 2018/848 [22]. These aims of the organic system correspond with the goals of the United Nations Environment Programme 2030 Agenda for Sustainable Development [23,24,25].

It is also considered that organic agriculture is one of the agronomic systems aiming to ensure high quality and safety of raw materials and food products [26]. The pesticide exposure, linked to various human health risks, is one of the important concerns of food safety [27,28]. Available research suggests that exposure to many widely used pesticides can be reduced by eating organic instead of conventionally grown fruits and vegetables [29,30]. Considering the above, and the fact that the consumption of vegetables and fruits is currently widely promoted as one of the most important prevention strategies against obesity and a number of non-communicable diseases [31], searching for and examination of various aspects of organic methods of plant cultivation, as well as characterisation of nutritional and health-promoting qualities of organically produced vegetables, should be of interest to researchers and consumers.

The results of numerous primary research studies, summarised by a few large meta-analyses, showed higher concentrations of various antioxidants in organically compared with conventionally cultivated [29,32,33,34,35,36,37]. However, only a limited number of published studies focused on organic versus non-organic courgette. The available research on nutritional characterisation of different courgette varieties is also limited. The present study therefore aimed to analyse and to compare antioxidants’ profiles and content in the fruits of different courgette varieties grown in three consecutive years in the organic and conventional production systems.

## 2. Materials and Methods

### 2.1. Study Design and Plant Material

In this study courgettes of two varieties, Astra Polka and Nimba, were cultivated in three consecutive years (2016, 2017, 2018) under organic and conventional management in a controlled trial established on the Experimental Field of the Department of Agronomy, Warsaw University of Life Sciences, in Miedniewice, Poland (51°57′ N 20°11′ E). The organic plots were certified by “AGRO BIO TEST” certification body since 2015.

Commercially available seeds of both varieties were purchased in a retail store in Poland. Certified organic seeds were produced by PlantiCo^®^ company (Zielonki Parcela, Poland) [38] and conventional seeds were produced by PNOS^®^ company (Ożarów Mazowiecki, Poland) [39].

Astra Polka and Nimba varieties produce fruits with similar morphological characteristics, such as long, cylindrical shape, slightly narrower at the stem, thin and dark green skin with light green pattern (Nimba) or light green tiny spots (Astra Polka), and creamy flesh.

Each cultivation system (organic and conventional) was established in a rectangular area of 123 m^2^, divided into 8 plots. Seeds of each of the two varieties were sown in 4 randomly selected replicate plots, with row spacing of 120 cm and with 80 cm distance between seeds within a row. In the effect, there were 16 plants (4 × 4) in each of the 8 plots (4 replicate plots × 2 varieties), giving 128 plants in the cultivation system. The seeds were hand sown into the soil to a depth of approximately 3 times the size of the seed, after spring frosts (sowing dates: 17 May 2016, 26 May 2017, 18 May 2018).

The research field in Miedniewice is characterised by lessive soil, created by the mix of loamy sand and light loam. Different crop rotations were used in both cultivation systems in the years preceding the experiment. The information about crop rotations and fertilisation used are presented in Table 1. Courgette followed winter spelt in the organic rotation, and spring barley in the conventional rotation. Neither weed nor pest and disease protection were applied in the experiment. The choice of organic and mineral fertilizers and the nitrogen, phosphorus, and potassium (NPK) level was calculated from conversion factors based on Siebeneicher’s recommendations [40].

Fruits were harvested at technological maturity stage, three times in every harvest season: in the beginning of July, at the turn of July and August, and at the end of August.

**Figure 1:**
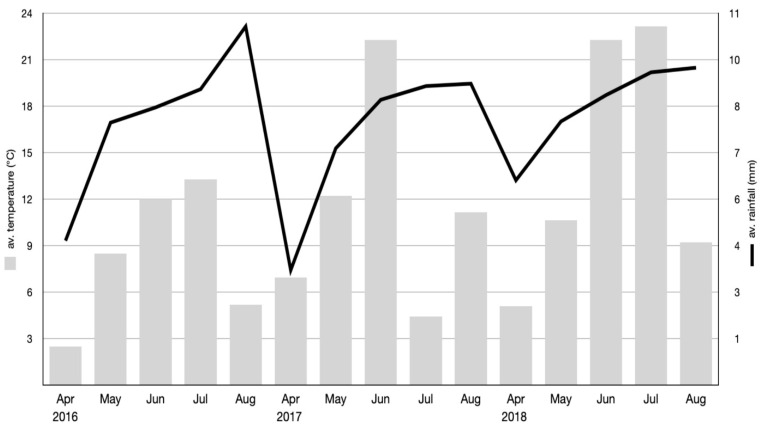
Weather conditions (temperatures and rainfall) in vegetation seasons of all three years of courgette cultivation.

Weather conditions (temperatures and rainfall) in vegetation season of all three experimental years are shown in Figure 1.

### 2.2. Preparation of Samples

The random fruit samples of 5 kg were collected from each of the 4 organic and 4 conventional plots and transported to the laboratory of the Department of Functional and Organic Food. Fruits were washed and cut into cubes (5 × 5 × 5 mm). The 10–15 g fresh fruit samples were used for dry matter content analysis. The 100 g samples were frozen in −80 °C for 24 h and freeze-dried using Labcono 2.5 freeze-dryer (Labconco Corporation, Kansas City, MO, USA), under the temperature of −40 °C and the pressure of 0.100 mBa. Dried samples were ground in laboratory mill and stored in scintillation vials in −80 °C temperature before further analyses of polyphenols (phenolic acids and flavonoids), carotenoids (lutein, zeaxanthin, β-carotene), vitamin C (dehydroascorbic acid, l-ascorbic acid), and chlorophylls (*a*, *b*) content.

### 2.3. Chemicals

Acetone (HPLC grade) came from Sigma-Aldrich (Poznań, Poland); Acetonitrile (HPLC grade) came from Chempur (Piekary Śląskie, Poland); Carotenoids, vitamin C, chlorophylls, and phenolics standards (HPLC grade 99.5–99.9% pure) came from Fluka and Sigma-Aldrich (Poznań, Poland): β-carotene, caffeic acid, chlorogenic acid, chlorophyll *a*, chlorophyll *b*, dehydroascorbic acid, ferulic acid, gallic acid, kaempferol-3-*O*-glucoside, lutein, l-ascorbic acid, *p*-coumaric acid, quercetin, quercetin-3-*O*-rutinoside; Ethyl acetate came from Sigma Aldrich (Poznań, Poland); Magnesium carbonate (ultrapure) came from Chempur (Piekary Śląskie, Poland); Meta-phosphoric acid came from Sigma-Aldrich (Poznań, Poland); Methanol (HPLC grade) came from Chempur (Piekary Śląskie, Poland); Ortho-phosphoric acid came from Chempur (Piekary Śląskie, Poland).

### 2.4. Dry Matter

Dry matter content was assessed using standard procedure (Polish Norm PN-EN-12145:2001) [42]. Fresh samples were dried in the temperature of 105 °C with free air circulation for 72 h (FP-25W Farma Play dryer, Farma Play, Marki, Poland). Dried samples were cooled in a desiccator and weighed. Dry matter content was calculated in percent of fresh material.

### 2.5. Phenolic Compounds Extraction and Identification

Phenolic compounds in the courgette samples were determined by High Performance Liquid Chromatography (HPLC), as previously described by Hallmann et al. [43]. One hundred milligrams of freeze-dried courgette powder was used to prepare extract from every sample. The material was mixed with 5 mL of 80% methanol (*v*/*v* aqueous solution) and shaken in a Micro-Shaker 326 M (Poland). Then it was placed in an ultrasonic bath (10 min, 30 °C, 5500 Hz) [44]. Next, the sample was centrifuged (10 min, 3780 g, 5 °C). The decanted supernatant was re-centrifuged (5 min, 31,180× *g*, 0 °C), collected into vials and analysed by HPLC device (Shimadzu, USA Manufacturing, Inc., Canby, OR, USA: two pumps LC-20AD, controller CBM-20A, column oven SIL-20AC, spectrometer UV/Vis SPD-20 AV). The injection volume was 100 µL. For polyphenol compounds separation and identification, a Synergi Fusion-RP 80i Phenomenex column (250 × 4.60 mm) was used. The phenolic compounds were separated under gradient conditions with a flow rate of 1 mL min^−1^. Two gradient phases were used, 10% (*v*/*v*) acetonitrile and ultra-pure water (phase A) and 55% (*v*/*v*) acetonitrile and ultrapure water (phase B). The phases were acidified by ortho-phosphoric acid (pH 3.0). The wavelength used for detection was 270–360 nm. For compounds identification, the external standards of polyphenols with purities of 95.00–99.99% were used. The concentrations of polyphenols were calculated based on standard curve and sample dilution coefficients.

### 2.6. Carotenoids and Chlorophylls Extraction and Identification

Carotenoids and chlorophylls were determined using High Performance Liquid Chromatography (HPLC), following the methodology described by Nishiyama et al. [45] with slight modifications. A 100 mg sample of freeze-dried courgette fruit powder was mixed by Vortex mixer with 5 mL of pure acetone. Next, samples were incubated in ultrasonic bath (15 min, 0 °C) and centrifuged (6000 rpm, 10 min, 0 °C). The sonication conditions applied were based on the study of Singh et al. [46]. The supernatant was transferred to dark HPLC vials. The previously described Shimadzu equipment (two LC-20AD pumps, CMB-20A system controller, SIL-20AC autosampler, UV-Vis SPD-20AV detector, Shimadzu, USA Manufacturing Inc., Canby, OR, USA was used. A Max-RP 80A column (250 × 4.6 mm) was used for the compounds separation. The wavelength used was 445–450 nm. Time of the analysis was 18 min. The injection volume was 100 µL. The chromatographic peaks corresponding to particular carotenoids and chlorophylls were identified by comparing the retention times with those of authentic standards (Fluka and Sigma-Aldrich, Poznań, Poland, purity of 99.98%). For confirmation, co-chromatography of each sample with the standard was also applied [43].

### 2.7. Vitamin C Extraction and Identificiation

The analysis of l-ascorbic acid (l-ASC) and dehydroascorbic acid (DHA) was performed using High Performance Liquid Chromatography (HPLC) method. As a solvent, 2 mL of 5% meta-phosphoric acid were added to the sample. Then, sample was mixed by vortex mixer (5 seconds), incubated in an ultrasonic bath (15 min, 20 °C) and centrifuged (6000 rpm, 0 °C). The supernatant (1mL) was transferred to dark HPLC vials. The Phenomenex Hydro 80-A RP column (250 × 4.6 mm), the mobile phase of 50 mM phosphate buffer (pH 4.4), and 0.1 mM sodium acetate were used for analysis. Time of the analysis was 18 min, and the detection wavelength was 255–260 nm. The compounds were identified based on the retention time of the external Sigma-Aldrich l-ASC and DHA standards. The applied extraction and analytical method was based on the study of Nojovan et al. [47], optimised based on Van de Velde et al. [48], with our own modifications [49].

### 2.8. Statistical Analysis 

The statistical analyses were carried out in the R statistical environment [50]. The 3-factor Analyses of Variance (ANOVA) were performed using a linear-mixed effects model, with year, courgette variety, and cultivation system as fixed effects, and field replication as a random effect factor. This allowed to test the significance of the experimental factors and their interactions. When 3-factor interactions were identified, the additional 2-factor ANOVA was carried out, to explain the interactions. The significance of differences between the interaction means was tested using the Tukey’s HSD post hoc test. Before the analyses, the normality of data distribution was verified using the qqnorm function in R, and data transformation was used when necessary. The cube root transformation was applied to data on the concentration of chlorophyll *a*, dehydroascorbic acid (DHA), ferulic acid, flavonoids (sum), gallic acid, *p*-coumaric acid, and quercetin-3-*O*-rutinoside. The logarithmic transformation was applied to data on β-carotene, phenolic acids (sum), kaempferol-3-*O*-glucoside, carotenoids (sum), lutein, phenolic acids (sum), and dry matter. Square root transformation was applied to data on chlorophyll *b*, caffeic acid, l-ascorbic acid (l-ASC), and vitamin C (total). Data on the content of chlorophylls (sum) and chlorogenic acid were normally distributed and did not require transformation.

## 3. Results

### 3.1. Dry Matter Content

The average dry matter content in the courgette fruits analysed within the study was 5.09 ± 0.08 g/100 g f.w. The production system significantly affected dry matter content in courgette fruits. Organic courgettes had more dry matter than conventionally cultivated fruits. Statistical analysis showed also significant differences of average dry matter content between years of cultivation. The highest dry matter content was found in fruits cultivated in 2017, while the lowest in fruits grown in 2018. No significant effect of variety on the dry matter content in courgette fruits was found (Table 2, Table S1 and Figure S1 in Supplementary Material).

### 3.2. Vitamin C Content

The average content of vitamin C in the courgette fruits analysed in this experiment reached 6.17 ± 0.20 mg/100 g f.w. The study has shown that l-ascorbic acid and the vitamin C (sum of l-ASC and DHA) content in courgette fruits was not influenced by the cultivation system. Only the significant impact of the system on DHA was observed. Its content was found to be higher in conventional fruits. The nature of the effect of cultivation year on l-ASC and DHA content was opposite: Fruits in 2018 had significantly highest concentration of l-ASC but the lowest concentration of DHA. No interactions between factors on the concentration of vitamin C (total), DHA, and l-ASC were noted (Table 2, Figures S2–S4 in Supplementary Material).

### 3.3. Phenolic Acids Content

The summary content of phenolic acids identified in the courgette fruits within the study reached, on average, 29.67 ± 1.11 µg/g f.w., which accounted to over 90% of all identified polyphenols (32.78 ± 1.31 µg/g f.w.). Gallic and *p*-coumaric acid were the most abundant among all phenolic acids detected in the courgette fruits (12.37 ± 0.54 µg/g f.w. and 8.00 ± 0.25 µg/g f.w., respectively). The production system had a significant impact on the concentration of all identified phenolic acids. The higher content of these compounds was found in organic compared to the conventionally cultivated plants. Similarly, the significant effect of the growing season (year) was also observed for all compounds. In case of most of the compounds, their concentrations were higher in 2017, while lower in years 2016 and 2018. No effect of variety on the content of phenolic acids in courgette fruits was detected. At the same time, significant interactions between the effects of agronomic system and cultivation year on the concentrations of most of the identified phenolic acids were identified (Table 3 and Table S2, Figures S5–S12 in Supplementary Material): chlorogenic and ferulic acid content were higher in organic than in conventional courgettes in 2017 and 2018, but no effect of cultivation system was found in 2016. For caffeic acid a higher content in organic fruits was observed only in 2016 and 2018, while system had no effect in 2017 (Table 4). The same relationship as in chlorogenic and ferulic acids was found in *p*-coumaric acid, but only for the Astra Polka variety. In Nimba, the effect of agronomic system was noted only in 2018, where organic cultivation was associated with higher content of this phenolic compound (Table 5).

### 3.4. Flavonoids Content

The average content of flavonoids (sum) in the courgette fruits analysed within the study reached 3.11 ± 0.22 µg/g f.w. The production system and year of cultivation had a significant impact on the concentrations of each of the identified flavonoids, quercetin-3-*O*-rutinoside and kaempferol-3-*O*-glucoside, in the courgette fruits (Table 2; Table 3). The quercetin-3-*O*-rutinoside and kaempferol-3-*O*-glucoside were found in higher concentrations in organic than in conventional courgette, and their highest contents were noted in 2017. However, two-way interactions between year and cultivation system on kaempferol-3-*O*-glucoside and flavonoids (sum) were found: the content of kaempferol-3-*O*-glucoside, as well as the sum of detected flavonoids, were significantly higher in organic than in conventional fruits in 2017 and 2018, but no impact of production system was found in 2016 (Table 4, Tables S1 and S2 and Figures S7, S13 and S14 in Supplementary Material).

### 3.5. Carotenoids Content

The average content of carotenoids (sum) in the courgette fruits analysed in this experiment reached 0.87 ± 0.03 mg/100 g f.w., and was dominated by β-carotene (0.76 ± 0.03 mg/100 g f.w.). The production system significantly impacted the carotenoids (sum), β-carotene and lutein content in plants and had no effect on zeaxanthin. Higher concentrations of carotenoids were found in organic compared to the conventional courgette fruits, except for zeaxanthin which was not affected by the agronomic system (Table 6). In addition, the significant impact of the cultivation year on carotenoids content was identified. Lutein concentration in 2018 was the lowest across all three seasons. In contrast, β-carotene content was found to be in the lowest level in 2016. The two-way interactions between the year and cultivation system were also found for β-carotene and lutein: The higher β-carotene and lutein content was detected in organic than in conventional fruits in 2017 and 2016, respectively; however, the samples in 2016 and 2018 (β-carotene) and 2017 and 2018 (lutein) were not affected by the cultivation system (Table 4, Table S3 and Figures S15–S18 in Supplementary Material).

### 3.6. Chlorophylls Content

The courgette fruits analysed within the study contained on average 2.31 ± 0.05 mg/100 g f.w. chlorophylls, with a predominant share of chlorophyll *a* (1.75 ± 0.04 mg/100 g f.w.). The production system and year of cultivation had an effect on the chlorophyll *a* and *b* content in courgette fruits. Both chlorophylls content was significantly greater in organic fruits compared to the conventional ones. The average concentrations of both chlorophylls were lowest in 2018. Moreover, the two- and three-way interactions between factors were found (Table 6, Table S3 and Figures S19–S21 in Supplementary Material): The significantly higher chlorophyll *a* content was found in organic fruits of Nimba variety in 2016 and organic fruits of Astra Polka variety in 2018, whereas no significant differences between organic and conventional fruits were noted in 2016 and 2017 (Astra Polka variety), as well as in 2017 and 2018 (Nimba variety). The organic fruits of Nimba were significantly more abundant in chlorophyll *b* in 2016 only, whereas no differences were noted in 2017 and 2018. There was no impact of the production system in all cultivation years in Astra Polka plants. The profile of chlorophylls (sum) was similar as chlorophyll *a* for both varieties and all years of the study (Table 5).

## 4. Discussion

Vegetables and fruits are recommended in the prevention of diseases linked to oxidative stress, because most of them are rich in bioactive compounds with strong antioxidant properties. Among them is courgette, frequently consumed worldwide. However, antioxidants profile of courgette, compared to other species of vegetables from the *Cucurbitaceae* family, is still not sufficiently recognised [20]. According to published research, quantitative and qualitative profile of antioxidants in courgette fruits may strongly depend on the variety, harvest period, growing conditions, and the stage of ripening [9].

In this study, the year of cultivation had the strongest effect on the level of bioactive compounds in courgette. The content of both DHA and vitamin C, but not l-ASC, were the highest in 2018. The content of almost all polyphenols analysed in this study was the lowest in 2016 and the highest in 2017. The only two exceptions were *p*-coumaric acid and caffeic acid, with the highest level in 2016 and 2018, respectively. Similar to polyphenols, the β-carotene, and the sum of carotenoids, also showed the lowest concentration in 2016. Lutein and zeaxanthin showed the lowest level in 2018, the same way as all chlorophylls.

Weather conditions in 2016 were characterised by the highest average temperature and very low average rainfall in August, while the highest rainfall was observed in July 2018. The lowest concentration of bioactive compounds in 2016, mainly phenolic acids, might be in line with the lowest rainfall in this year. The average rainfall and temperature in 2017 were within optimal level, which seems to be in line with the highest concentration of the analysed compounds.

Conventionally cultivated courgette was analysed already, and results showed similar quality and quantity of polyphenols as in this study. A *p*-coumaric acid was also pointed to as the main polyphenol in courgette, which is in line with the presented results [51]. However, very limited number of studies investigated the impact of cultivation system on the antioxidants content in courgette. Some studies have reported that the profile of phenolic acids and flavonoids can differ between organic and conventionally cultivated fruits [52]. The authors of another study showed that total phenolics content in the fruits significantly decreased with the increase of mineral NPK fertilisation level, which confirms presented results [9]. In addition, a higher level of chlorophylls content in organic vs. conventional fruits was detected by the other authors. The authors explain it by relation of chlorophyll and photosynthesis processes and increasing chlorophyll synthesis during oxidative stress [53]. The same trend with higher chlorophylls content in organic courgette was observed in our study. According to chemical structure of the chlorophyll (four nitrogen atoms and magnesium atom in the centre), the easily available nitrogen, characteristic for the conventional cultivation method, could effect in increasing of the chlorophyll content in the crop, which could be modulated by the availability and quantity of magnesium in soil. Studies about chlorophyll concentrations also suggest that light manipulations and mycorrhizal inoculation may affect the chlorophyll content in edible plants [54,55,56].

Many research studies pointed that organic farming methods may result in higher concentration of bioactive compounds in plants [36,57,58]. Higher content of vitamin C in organic fruits was also found. The researchers explained that activation of a secondary metabolism is observed in organic fruits as the reaction to numerous stress factors and to the limited nitrogen availability. It leads to a higher amount of vitamin C [59]. However, the research outcomes are not consistent in this respect—in other study, lower concentration of β-carotene and vitamin C in organic (cultivated with cow manure) than in conventional fruits was noted [60].

In the presented study, no significant effect of courgette variety on the detected antioxidants, expect for DHA, was found. However, the interaction of the production system and year with the variety effects on the chlorophylls *a* and *b*, lutein, β-carotene, and *p*-coumaric acid contents show different vulnerability of each of the tested courgette varieties to the cultivation method. Despite the individual characteristics of each variety, other authors showed slight differences between the number of bioactive compounds of two varieties of courgette (Verde and Redondo) [52]. Higher sensitivity to organic cultivation method may be a characteristic of several varieties of courgette, and this aspect was confirmed in this paper. According to other studies, the content of phenolic acids and flavonoids in courgette fruit can differ between courgette varieties [52].

## 5. Conclusions

Consumers are increasingly searching for high quality foods, rich in health-promoting antioxidants. Thus, investigating the impact of potential quality-modulating triggers, such as agronomic practices, especially in connection with plant genotype, is of importance. The presented study draws attention to courgette as an important source of health-promoting phytochemicals, such as phenolic acids, flavonoids, vitamin C, and carotenoids. It shows a significant variation in the content of selected groups of antioxidants between courgette fruits grown in organic and conventional system, underlying at the same time some significant interactions between the cultivation system and plant varieties. It is worth pointing that, despite the large year-to-year differences in the courgette fruit composition, the vast majority of the measured phenolics shown higher concentrations in organic compared to the conventional courgette fruits, and these trends were similar for both tested varieties. This suggests that organic cultivation might be a good method to increase concentration of these bioactive compounds with antioxidant properties in courgette fruits. Considering the very limited number of similar studies on courgette, these results seem to give the very first insights into the characteristics of courgette fruits grown in extensive, organic systems and could thus be of interest for the producers and the consumers. Nevertheless, the identified trends should be further confirmed, with attention being paid to other courgette varieties, and to the potential interactions between the plant genotype, agronomic system, and the location-specific growing conditions, to validate the conclusions.

## Figures and Tables

**Table 1:** Agronomic inputs and fertilisation in organic and conventional systems.

Crop Production Practice	Organic	Conventional		
Crop rotation	sugar beet + sheep manure-> winter spelt undersown with red clover-> red clover-> winter spelt	winter wheat-> winter rape-> spring barley		
Fertilizer (dose)	Sheep manure (20t/ha)	Polifoska (625 kg/ha)	Ammonium sulphate (219 kg/ha)	Superphosphate (263 kg/ha)
The content of main compounds in fertilizers (%)	N (total): 0.76 P_2_O_5_: 0.4 K_2_O: 1.25	N (total): 8 P_2_O_5_^2^: 24 P_2_O_5_^1^: 21 K_2_O ^1^: 24 SO_3_: 9	N-NH: 17.2 N-NO: 17.2	P_2_O_5_: 40 CaO: 10 Si: 5
Sum of N, P and K applied to soil (kg/ha) [41]	N: 45.6	N-NH: 37.7, N-NO: 37.7		
	P: 13.9	P: 124.6		
	K: 166.7	K: 125		

^1^ soluble in water; ^2^ soluble in neutral ammonium citrate and water.

**Table 2:** The main effects of, and interactions between, cultivation year, variety and agronomic system on the content of dry matter, vitamin C, and selected groups of phenolic compounds in courgette fruits.

Factor	Dry Matter ^1^	DHA ^2^	l-ASC ^3^	Vitamin C ^4^	Polyphenols (Sum) ^5^	Phenolic Acids (Sum) ^5^	Flavonoids (Sum) ^5^
Cultivation Year (CY)							
2016	5.29 ± 0.12 ^6^ b ^7^	0.88 ± 0.07 b	3.73 ± 0.19 a	4.61 ± 0.22 c	19.18 ± 0.85 c	18.44 ± 0.83 c	0.73 ± 0.03 c
2017	5.77 ± 0.13 a	1.31 ± 0.07 b	4.40 ± 0.32 a	5.70 ± 0.35 b	45.74 ± 2.70 a	40.69 ± 2.25 a	5.05 ± 0.48 a
2018	4.14 ± 0.10 c	7.02 ± 0.32 a	1.36 ± 0.10 b	8.37 ± 0.32 a	33.34 ± 1.81 b	29.76 ± 1.54 b	3.58 ± 0.32 b
Variety (VR)							
Astra Polka	5.06 ± 0.11	2.78 ± 0.28	3.14 ± 0.20	5.91 ± 0.29	32.76 ± 1.78	29.76 ± 1.52	3.00 ± 0.29
Nimba	5.13 ± 0.11	3.14 ± 0.28	3.31 ± 0.23	6.45 ± 0.27	32.81 ± 1.94	29.58 ± 1.62	3.23 ± 0.34
Agronomic System (AS)							
conventional	4.83 ± 0.10	3.12 ± 0.26	3.22 ± 0.18	6.34 ± 0.25	23.51 ± 1.02	21.44 ± 0.88	2.07 ± 0.16
organic	5.37 ± 0.13	2.77 ± 0.31	3.22 ± 0.25	5.99 ± 0.31	42.63 ± 2.17	38.40 ± 1.80	4.22 ± 0.40
ANOVA *p*-values							
CY	<0.001	<0.001	<0.001	<0.001	<0.001	<0.001	<0.001
VR	NS ^8^	0.002	NS	NS	NS	NS	NS
AS	0.003	0.020	NS	NS	<0.001	<0.001	<0.001
CY × VR	NS	NS	NS	NS	NS	NS	NS
CY × AS	NS	NS	NS	NS	NS	NS	0.012
VR × AS	NS	NS	NS	NS	NS	NS	NS
CY × VR × AS	NS	NS	NS	NS	NS	NS	NS

^1^ g/100 g f.w.; ^2^ Dehydroascorbic acid (DHA) (mg/100 g f.w.); ^3^ l-Ascorbic acid (l-ASC) (mg/100 g f.w.); ^4^ mg/100 g f.w.; ^5^ µg/g f.w.; ^6^ data are presented as means ± standard errors; ^7^ values in the same column followed by different letters (a–c) are significantly different at the 5% level of probability, with “a” always representing the highest value; ^8^ not significant (NS).

**Table 3:** The main effects of, and interactions between, cultivation year, variety, and agronomic system on the content of individual phenolic acids and flavonoids (µg/g f.w.) in courgette fruits.

Factor	Gallic Acid	Chlorogenic Acid	Caffeic Acid	*p*-Coumaric Acid	Ferulic Acid	Quercetin-3-*O*-Rutinoside	Kaempferol-3-*O*-Glucoside
Cultivation Year (CY)							
2016	6.02 ± 0.56 ^1^ c ^2^	0.53 ± 0.03 c	2.26 ± 0.31 a	9.15 ± 0.36 a	0.47 ± 0.02 c	0.22 ± 0.02 c	0.51 ± 0.01 c
2017	18.82 ± 0.96 a	6.98 ± 0.42 a	2.91 ± 0.24 a	8.22 ± 0.49 a	3.76 ± 0.37 a	2.88 ± 0.29 a	2.16 ± 0.20 a
2018	12.16 ± 0.62 b	5.57 ± 0.30 b	3.09 ± 0.37 a	6.48 ± 0.37 b	2.65 ± 0.24 b	1.98 ± 0.18 b	1.63 ± 0.15 b
Variety (VR)							
Astra Polka	12.55 ± 0.71	4.15 ± 0.35	2.62 ± 0.24	8.19 ± 0.37	2.24 ± 0.24	1.64 ± 0.18	1.36 ± 0.12
Nimba	12.17 ± 0.82	4.50 ± 0.35	2.88 ± 0.27	7.80 ± 0.33	2.34 ± 0.24	1.74 ± 0.20	1.50 ± 0.14
Agronomic System (AS)							
conventional	8.50 ± 0.48	3.20 ± 0.23	1.63 ± 0.13	6.69 ± 0.28	1.42 ± 0.12	1.15 ± 0.11	0.92 ± 0.06
organic	16.47 ± 0.85	5.54 ± 0.42	3.93 ± 0.31	9.39 ± 0.38	3.21 ± 0.31	2.27 ± 0.24	1.97 ± 0.17
ANOVA *p*-values							
CY	<0.001	<0.001	0.011	<0.001	<0.001	<0.001	<0.001
VR	NS ^3^	NS	NS	NS	NS	NS	NS
AS	<0.001	<0.001	<0.001	<0.001	<0.001	0.001	<0.001
CY × VR	NS	NS	NS	NS	NS	NS	NS
CY × AS	NS	<0.001	0.019	<0.001	0.001	NS	0.002
VR × AS	NS	NS	NS	NS	NS	NS	NS
CY × VR × AS	NS	NS	NS	0.007	NS	NS	NS

^1^ Data are presented as means ± standard errors; ^2^ values in columns followed by different letters (a–c) are significantly different at the 5% level of probability, with “a” always representing the highest value; ^3^ not significant.

**Table 4:** The main effects of, and interactions between, cultivation year and agronomic system on the content of chlorogenic acid, caffeic acid, ferulic acid, sum of flavonoids, kaempferol-3-*O*-glucoside, lutein, and β-carotene in courgette.

CY ^1^	AS ^2^	Chlorogenic Acid ^3^	Caffeic Acid ^3^	Ferulic Acid ^3^	Flavonoids (Sum) ^3^	Kaempferol-3-*O*-Glucoside ^3^	Lutein ^4^	β-Carotene ^4^
2016	conventional	0.43 ± 0.04 ^5^ c ^6^	0.63 ± 0.04 c	0.45 ± 0.03 d	0.66 ± 0.02 d	0.48 ± 0.02 c	0.100 ± 0.003 b,c	0.50 ± 0.02 b
2016	organic	0.64 ± 0.05 c	3.89 ± 0.53 a	0.49 ± 0.02 d	0.81 ± 0.04 d	0.55 ± 0.02 c	0.115 ± 0.004 a	0.62 ± 0.03 b
2017	conventional	5.27 ± 0.39 b	2.16 ± 0.21 b,c	2.21 ± 0.24 c	3.23 ± 0.33 c	1.32 ± 0.12 b	0.106 ± 0.003 a,b	0.60 ± 0.03 b
2017	organic	8.81 ± 0.67 a	3.72 ± 0.42 a,b	5.42 ± 0.64 a	6.98 ± 0.83 a	3.06 ± 0.34 a	0.100 ± 0.002 b,c	1.03 ± 0.04 a
2018	conventional	3.84 ± 0.24 b	2.11 ± 0.29 b,c	1.58 ± 0.16 c,d	2.27 ± 0.22 c,d	0.94 ± 0.08 b,c	0.083 ± 0.003 d	0.85 ± 0.08 a
2018	organic	7.69 ± 0.36 a	4.21 ± 0.68 a	3.86 ± 0.40 b	5.07 ± 0.54 b	2.40 ± 0.24 a	0.091 ± 0.003 c,d	1.06 ± 0.11 a
ANOVA *p*-values								
CY		<0.001	0.010	<0.001	<0.001	<0.001	<0.001	<0.001
AS		<0.001	<0.001	<0.001	<0.001	<0.001	0.013	<0.001
CY × AS		<0.001	0.016	0.001	0.011	0.002	0.018	0.037

^1^ Cultivation year; ^2^ agronomic system; data for varieties were averaged; ^3^ µg/g f.w.; ^4^ mg/100 g f.w.; ^5^ data are presented as means ± standard errors; ^6^ values in the same column followed by different letters (a–d) are significantly different at the 5% level of probability, with “a” always representing the highest value.

**Table 5:** The main effects of, and interactions between, cultivation year and agronomic system on the content of *p*-coumaric acid, sum of chlorophylls, chlorophyll *a*, and chlorophyll *b* in two varieties of courgette.

CY ^1^	AS ^2^	*p*-Coumaric Acid ^3^	Chlorophylls (Sum) ^4^	Chlorophyll *b* ^4^	Chlorophyll *a* ^4^
Variety Astra Polka					
2016	conventional	10.48 ± 0.54 ^5^ a ^6^	2.34 ± 0.11 b, c	0.55 ± 0.03 a,b	1.79 ± 0.10 a,b
2016	organic	8.77 ± 0.91 a	2.78 ± 0.12 a, b	0.62 ± 0.03 a	2.16 ± 0.10 a
2017	conventional	5.55 ± 0.54 b	2.46 ± 0.14 a,b,c	0.60 ± 0.02 a,b	1.86 ± 0.13 a, b
2017	organic	11.09 ± 1.18 a	2.87 ± 0.11 a	0.62 ± 0.03 a,b	2.22 ± 0.09 a
2018	conventional	3.92 ± 0.38 b	1.62 ± 0.10 d	0.44 ± 0.02 c	1.17 ± 0.08 c
2018	organic	9.51 ± 0.64 a	2.19 ± 0.15 c	0.52 ± 0.03 b,c	1.67 ± 0.13 b
Variety Nimba					
2016	conventional	8.30 ± 0.55 a	1.97 ± 0.09 c	0.48 ± 0.02 b	1.48 ± 0.07 b
2016	organic	8.96 ± 0.79 a	2.99 ± 0.13 a	0.68 ± 0.03 a	2.31 ± 0.12 a
2017	conventional	7.01 ± 0.57 a,b	2.14 ± 0.11 b,c	0.59 ± 0.03 a,b	1.55 ± 0.09 b
2017	organic	9.45 ± 1.12 a	2.56 ± 0.18 a,b	0.64 ± 0.03 a	1.92 ± 0.16 a,b
2018	conventional	4.65 ± 0.31 b	1.98 ± 0.16 c	0.49 ± 0.03 b	1.49 ± 0.13 b
2018	organic	8.36 ± 0.79 a	1.94 ± 0.13 c	0.50 ± 0.03 b	1.44 ± 0.11 b
ANOVA *p*-values					
CY		0.002	<0.001	<0.001	<0.001
AS		0.016	0.004	0.008	0.006
CY × AS		NS ^7^	<0.001	0.004	0.003

^1^ Cultivation year; ^2^ Agronomic system; ^3^ µg/g f.w.; ^4^ mg/100 g f.w.; ^5^ data are presented as means ± standard errors; ^6^ values in the same column followed by different letters (a–c) are significantly different at the 5% level of probability, with “a” always representing the highest value; ^7^ not significant.

**Table 6:** The main effects of, and interactions between, cultivation year, variety, and agronomic system on the content of carotenoids and chlorophylls (mg/100 g f.w.) in courgette fruits.

Factor	Carotenoids (Sum)	Lutein	Zeaxanthin	β-Carotene	Chlorophylls (Sum)	Chlorophyll *b*	Chlorophyll *a*
Cultivation Year (CY)							
2016	0.66 ± 0.02 ^1^ b ^2^	0.107 ± 0.003 a	-	0.56 ± 0.02 b	2.53 ± 0.07 a	0.59 ± 0.01 a	1.94 ± 0.06 a
2017	0.94 ± 0.04 a	0.103 ± 0.002 a	0.036 ± 0.001	0.80 ± 0.04 a	2.50 ± 0.07 a	0.61 ± 0.01 a	1.89 ± 0.06 a
2018	1.05 ± 0.07 a	0.087 ± 0.002 b	0.027 ± 0.001	0.95 ± 0.06 a	1.93 ± 0.07 b	0.49 ± 0.01 b	1.44 ± 0.06 b
Variety (VR)							
Astra Polka	0.88 ± 0.04	0.099 ± 0.002	0.031 ± 0.001	0.77 ± 0.03	2.34 ± 0.06	0.55 ± 0.01	1.78 ± 0.05
Nimba	0.86 ± 0.04	0.099 ± 0.002	0.031 ± 0.001	0.75 ± 0.04	2.28 ± 0.07	0.56 ± 0.01	1.71 ± 0.06
Agronomic System (AS)							
conventional	0.76 ± 0.03	0.095 ± 0.002	0.030 ± 0.001	0.65 ± 0.03	2.06 ± 0.05	0.52 ± 0.01	1.54 ± 0.04
organic	0.99 ± 0.04	0.103 ± 0.002	0.032 ± 0.001	0.87 ± 0.04	2.57 ± 0.07	0.60 ± 0.01	1.97 ± 0.06
ANOVA *p*-values							
CY	<0.001	<0.001	<0.001	<0.001	<0.001	<0.001	<0.001
VR	NS ^3^	NS	NS	NS	NS	NS	NS
AS	<0.001	0.015	NS	<0.001	<0.001	<0.001	<0.001
CY × VR	NS	NS	NS	NS	NS	NS	NS
CY × AS	NS	0.020	NS	0.039	0.036	0.026	NS
VR × AS	NS	NS	NS	NS	NS	NS	NS
CY × VR × AS	NS	NS	NS	NS	0.003	0.028	0.006

^1^ Data are presented as means ± standard errors; ^2^ values in columns followed by different letters (a and b) are significantly different at the 5% level of probability, with “a” always representing the highest value; ^3^ not significant.

## Supplementary Materials

The following are available online at https://www.mdpi.com/2076-3921/9/5/404/s1.
