# Supplementary material for: The Profile of Selected Antioxidants in Two Courgette Varieties from Organic and Conventional Production
Source: Antioxidants (Basel). 2020 May 9;9(5):404. doi: 10.3390/antiox9050404 (PMC7278829; doi:10.3390/antiox9050404)
Supplement: Supplementary file 1 [file antioxidants-09-00404-s001.pdf]

Supplementary Material to the Article:

The profile of selected antioxidants in two courgette varieties from organic and conventional production

Klaudia Kopczyńska [1], Renata Kazimierczak [1], Dominika Średnicka-Tober [1,*], Marcin Barański [1], Zdzisław Wyszyński [2], Katarzyna Kucińska [2], Aneta Perzanowska [2], Paweł Szacki [2], Ewa Rembiałkowska [1] and Ewelina Hallmann [1]

[1] Department of Functional and Organic Food, Institute of Human Nutrition Sciences, Warsaw University of Life Sciences, Nowoursynowska 159c, 02-776 Warsaw, Poland; klaudia_kopczynska@sggw.edu.pl (K.K.); renata_kazimierczak@sggw.edu.pl (R.K.); marcin_baranski@sggw.edu.pl (M.B.); ewa_rembialkowska@sggw.edu.pl (E.R.); ewelina_hallmann@sggw.edu.pl (E.H.)

[2] Department of Agronomy, Institute of Agriculture, Warsaw University of Life Sciences, Nowoursynowska 159, 02-787 Warsaw, Poland; zdzislaw_wyszynski@sggw.edu.pl (Z.W.); katarzyna_kucinska@sggw.edu.pl (K.K.); aneta_perzanowska@sggw.edu.pl (A.P.); pawel_szacki@sggw.edu.pl (P.S.)

* Correspondence: dominika_srednicka_tober@sggw.edu.pl; Tel.: +48-22-593-7035

.

Table S1. The effects of cultivation year, variety and agronomic system on the content of dry matter, vitamin C and selected groups of phenolic compounds in courgette fruits.

CY	AS	dry matter [1]	DHA [2]	L-ASC [3]	vitamin C [4]	polyphenols (sum) [5]	phenolic acids (sum) [5]	flavonoids (sum) [5]
**Variety Astra Polka**								
2016	conventional	4.93 ± 0.22 [6] bc [7]	0.87 ± 0.14 b	3.09 ± 0.25 ab	3.96 ± 0.34 c	15.18 ± 0.69 d	14.52 ± 0.68 d	0.66 ± 0.03 c
2016	organic	5.56 ± 0.28 ab	0.72 ± 0.14 b	3.97 ± 0.46 a	4.69 ± 0.50 c	24.19 ± 1.59 cd	23.28 ± 1.54 cd	0.91 ± 0.06 c
2017	conventional	5.44 ± 0.15 abc	1.29 ± 0.18 b	4.13 ± 0.37 a	5.42 ± 0.53 bc	31.48 ± 2.14 c	28.20 ± 1.71 c	3.28 ± 0.47 b
2017	organic	6.05 ± 0.37 a	0.88 ± 0.08 b	4.40 ± 0.78 a	5.29 ± 0.80 bc	59.13 ± 6.22 a	52.92 ± 5.14 a	6.22 ± 1.13 a
2018	conventional	3.89 ± 0.16 d	6.76 ± 0.46 a	2.00 ± 0.26 bc	8.76 ± 0.53 a	21.71 ± 1.56 cd	19.35 ± 1.25 cd	2.36 ± 0.33 bc
2018	organic	4.49 ± 0.20 cd	6.43 ± 0.96 a	1.07 ± 0.12 c	7.51 ± 0.91 ab	46.49 ± 2.51 b	41.77 ± 2.06 b	4.73 ± 0.68 ab
**Variety Nimba**								
2016	conventional	4.86 ± 0.19 bc	1.02 ± 0.11 b	3.90 ± 0.34 a	4.92 ± 0.37 b	13.36 ± 0.80 d	12.69 ± 0.78 d	0.66 ± 0.04 d
2016	organic	5.80 ± 0.24 a	0.91 ± 0.17 b	4.01 ± 0.45 a	4.93 ± 0.52 b	23.92 ± 1.93 cd	23.21 ± 1.90 cd	0.71 ± 0.05 d
2017	conventional	5.71 ± 0.20 ab	1.87 ± 0.11 b	4.72 ± 0.58 a	6.58 ± 0.62 ab	34.60 ± 3.10 bc	31.41 ± 2.66 bc	3.18 ± 0.48 bc
2017	organic	5.88 ± 0.28 a	1.17 ± 0.09 b	4.33 ± 0.77 a	5.50 ± 0.82 b	59.45 ± 6.36 a	51.67 ± 5.17 a	7.78 ± 1.22 a
2018	conventional	3.99 ± 0.20 c	7.25 ± 0.40 a	1.31 ± 0.10 b	8.56 ± 0.43 a	23.86 ± 1.98 cd	21.69 ± 1.75 cd	2.17 ± 0.28 cd
2018	organic	4.18 ± 0.23 c	7.78 ± 0.54 a	0.93 ± 0.16 b	8.71 ± 0.53 a	43.94 ± 4.46 b	38.48 ± 3.65 b	5.46 ± 0.86 ab

[1] g/100g f.w.; [2] Dehydroascorbic acid (mg/100g f.w.); [3] L-Ascorbic acid (mg/100g f.w.); [4] mg/100g f.w.; [5] µg/g f.w.; [6] Data are presented as means ± standard errors; [7] Within each variety, values in the same column followed by different letters are significantly different at the 5 % level of probability, with 'a' always representing the highest value.

Table S2. The effects of cultivation year, variety and agronomic system on the content of phenolic acids and flavonoids (μg/g f.w.) in courgette fruits.

CY	AS	gallic acid	chlorogenic acid	caffeic acid	*p*-coumaric acid	ferulic acid	quercetin-3-*O*-rutinoside	kaempferol-3-*O*-glucoside
**Variety Astra Polka**								
2016	conventional	2.54 ± 0.27 [1] e [2]	0.38 ± 0.05 c	0.70 ± 0.06 b	10.48 ± 0.54 a	0.43 ± 0.04 c	0.16 ± 0.03 c	0.50 ± 0.03 c
2016	organic	10.10 ± 1.26 cd	0.60 ± 0.07 c	3.29 ± 0.62 a	8.77 ± 0.91 a	0.51 ± 0.04 c	0.30 ± 0.05 c	0.61 ± 0.04 c
2017	conventional	14.03 ± 0.74 bc	4.41 ± 0.35 b	2.15 ± 0.28 ab	5.55 ± 0.54 b	2.05 ± 0.32 bc	2.00 ± 0.33 b	1.28 ± 0.17 bc
2017	organic	23.83 ± 1.66 a	9.06 ± 1.13 a	3.62 ± 0.64 a	11.09 ± 1.18 a	5.32 ± 0.96 a	3.49 ± 0.68 a	2.72 ± 0.46 a
2018	conventional	8.65 ± 0.56 d	3.29 ± 0.22 b	2.00 ± 0.38 ab	3.92 ± 0.38 b	1.48 ± 0.23 c	1.44 ± 0.24 bc	0.92 ± 0.11 c
2018	organic	16.79 ± 0.99 b	7.45 ± 0.47 a	4.24 ± 0.99 a	9.51 ± 0.64 a	3.78 ± 0.51 ab	2.50 ± 0.38 ab	2.23 ± 0.32 ab
**Variety Nimba**								
2016	conventional	2.88 ± 0.33 d	0.48 ± 0.05 d	0.56 ± 0.06 c	8.30 ± 0.55 a	0.47 ± 0.05 d	0.21 ± 0.03 d	0.45 ± 0.02 b
2016	organic	8.65 ± 1.15 c	0.68 ± 0.08 d	4.45 ± 0.83 a	8.96 ± 0.79 a	0.47 ± 0.03 d	0.22 ± 0.04 d	0.49 ± 0.02 b
2017	conventional	13.76 ± 1.01 bc	6.12 ± 0.67 bc	2.17 ± 0.31 bc	7.01 ± 0.57 ab	2.36 ± 0.35 bc	1.81 ± 0.31 bc	1.37 ± 0.18 b
2017	organic	24.32 ± 2.64 a	8.56 ± 0.74 a	3.82 ± 0.57 ab	9.45 ± 1.12 a	5.53 ± 0.87 a	4.37 ± 0.73 a	3.41 ± 0.50 a
2018	conventional	8.68 ± 0.85 c	4.44 ± 0.40 c	2.23 ± 0.47 abc	4.65 ± 0.31 b	1.69 ± 0.23 cd	1.20 ± 0.18 cd	0.97 ± 0.11 b
2018	organic	15.30 ± 1.40 b	8.04 ± 0.56 ab	4.17 ± 0.96 ab	8.36 ± 0.79 a	3.95 ± 0.66 ab	3.03 ± 0.50 ab	2.60 ± 0.38 a

[1] Data are presented as means ± standard errors; [2] Within each variety, values in columns followed by different letters are significantly different at the 5 % level of probability, with 'a' always representing the highest value.

Table S3. The main effects of cultivation year, variety and agronomic system on the content of carotenoids and chlorophylls (mg/100g f.w.) in courgette fruits.

CY	AS	carotenoids (sum)	lutein	zeaxanthin	*β*-carotene	chlorophylls (sum)	chlorophyll *a*	chlorophyll *b*
**Variety Astra Polka**								
2016	conventional	0.63 ± 0.03 [1] c [2]	0.102 ± 0.005 ab	-	0.53 ± 0.03 c	2.34 ± 0.11 bc	1.79 ± 0.10 ab	0.55 ± 0.03 ab
2016	organic	0.77 ± 0.04 bc	0.115 ± 0.006 a	-	0.66 ± 0.04 bc	2.78 ± 0.12 ab	2.16 ± 0.10 a	0.62 ± 0.03 a
2017	conventional	0.80 ± 0.04 bc	0.105 ± 0.003 ab	0.036 ± 0.001 a	0.65 ± 0.04 bc	2.46 ± 0.14 abc	1.86 ± 0.13 ab	0.6 ± 0.02 ab
2017	organic	1.09 ± 0.05 ab	0.097 ± 0.003 ac	0.036 ± 0.002 a	0.96 ± 0.05 ab	2.87 ± 0.11 a	2.22 ± 0.09 a	0.62 ± 0.03 ab
2018	conventional	0.84 ± 0.08 bc	0.081 ± 0.003 c	0.025 ± 0.001 b	0.73 ± 0.08 bc	1.62 ± 0.10 d	1.17 ± 0.08 c	0.44 ± 0.02 c
2018	organic	1.22 ± 0.14 a	0.094 ± 0.005 bc	0.029 ± 0.001 b	1.15 ± 0.13 a	2.19 ± 0.15 c	1.67 ± 0.13 b	0.52 ± 0.03 bc
**Variety Nimba**								
2016	conventional	0.56 ± 0.03 c	0.097 ± 0.004 bc	-	0.47 ± 0.02 b	1.97 ± 0.09 c	1.48 ± 0.07 b	0.48 ± 0.02 b
2016	organic	0.69 ± 0.04 c	0.115 ± 0.005 a	-	0.58 ± 0.04 b	2.99 ± 0.13 a	2.31 ± 0.12 a	0.68 ± 0.03 a
2017	conventional	0.68 ± 0.03 bc	0.106 ± 0.005 ab	0.037 ± 0.002 a	0.54 ± 0.03 b	2.14 ± 0.11 bc	1.55 ± 0.09 b	0.59 ± 0.03 ab
2017	organic	1.23 ± 0.06 a	0.103 ± 0.004 abc	0.037 ± 0.002 a	1.09 ± 0.06 a	2.56 ± 0.18 ab	1.92 ± 0.16 ab	0.64 ± 0.03 a
2018	conventional	1.09 ± 0.13 a	0.084 ± 0.004 c	0.026 ± 0.001 b	0.98 ± 0.13 a	1.98 ± 0.16 c	1.49 ± 0.13 b	0.49 ± 0.03 b
2018	organic	1.08 ± 0.18 ab	0.088 ± 0.005 bc	0.027 ± 0.001 b	0.96 ± 0.18 a	1.94 ± 0.13 c	1.44 ± 0.11 b	0.5 ± 0.03 b

[1] Data are presented as means ± standard errors; [2] Within each variety, values in columns followed by different letters are significantly different at the 5 % level of probability, with 'a' always representing the highest value.

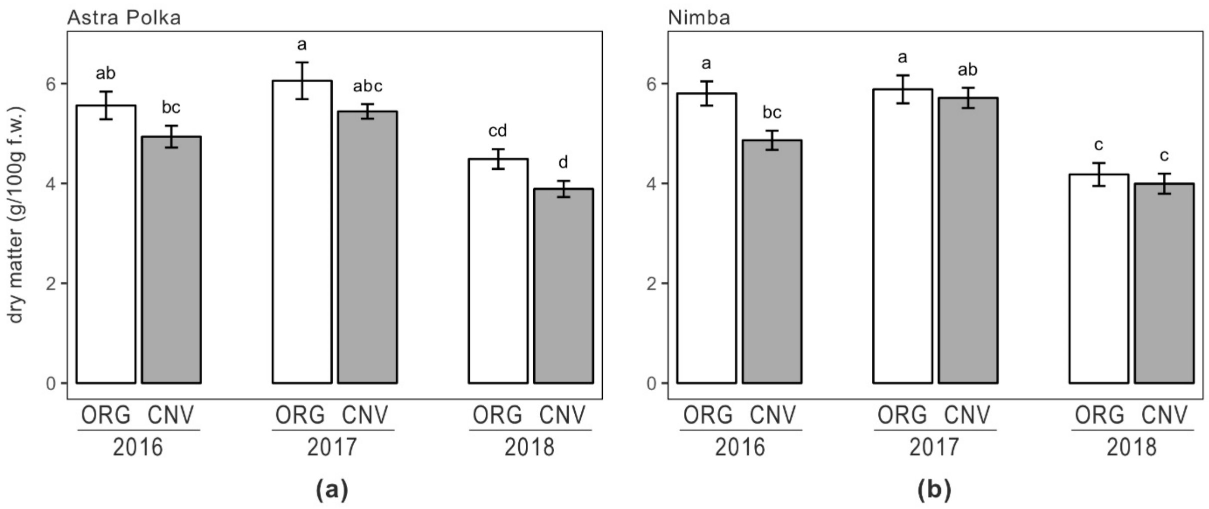

Figure S1. Dry matter in organic (ORG) and conventional (CNV) courgette fruits of (**a**) Astra Polka and (**b**) Nimba varieties in three years of cultivation. Data are presented as means with standard errors. Within each variety, bars marked with the same letters are not significantly different at the 5% level of probability.

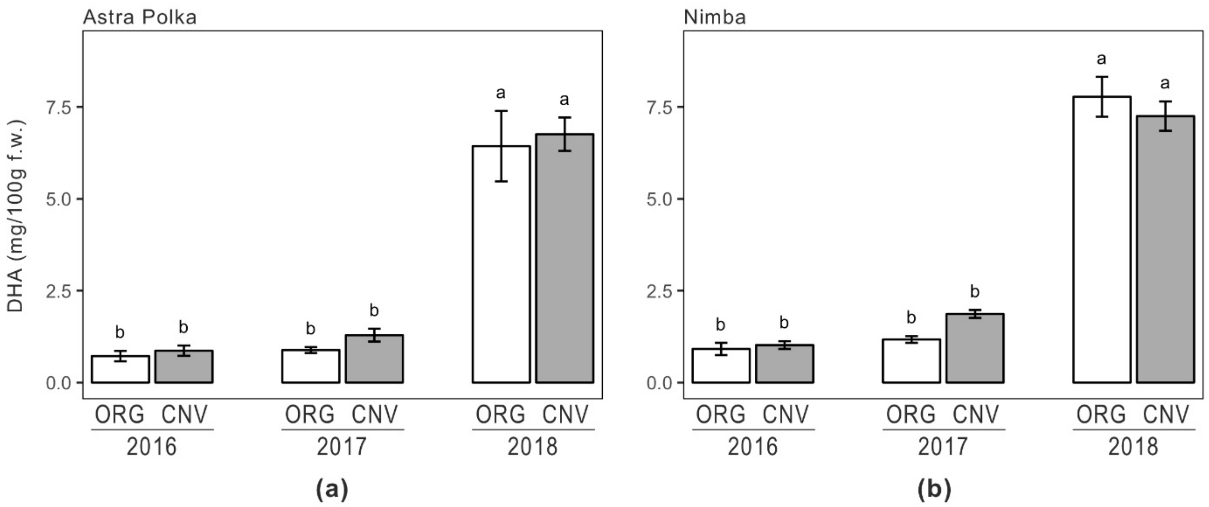

Figure S2. Dehydroascorbic acid (DHA) content in organic (ORG) and conventional (CNV) courgette fruits of (**a**) Astra Polka and (**b**) Nimba varieties in three years of cultivation. Data are presented as means with standard errors. Within each variety, bars marked with the same letters are not significantly different at the 5% level of probability.

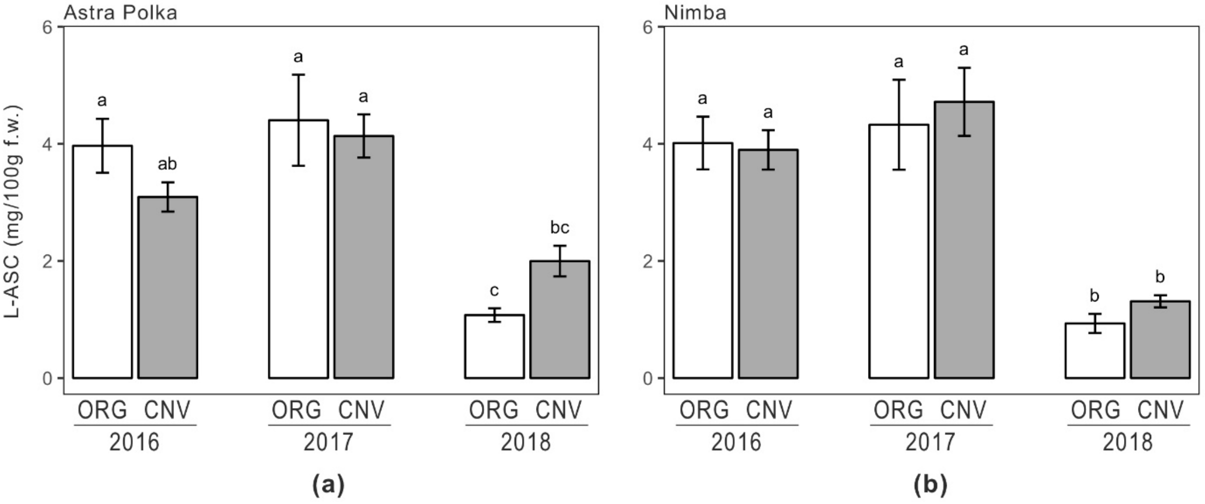

Figure S3. L-ascorbic acid (L-ASC) content in organic (ORG) and conventional (CNV) courgette fruits of (**a**) Astra Polka and (**b**) Nimba varieties in three years of cultivation. Data are presented as means with standard errors. Within each variety, bars marked with the same letters are not significantly different at the 5% level of probability.

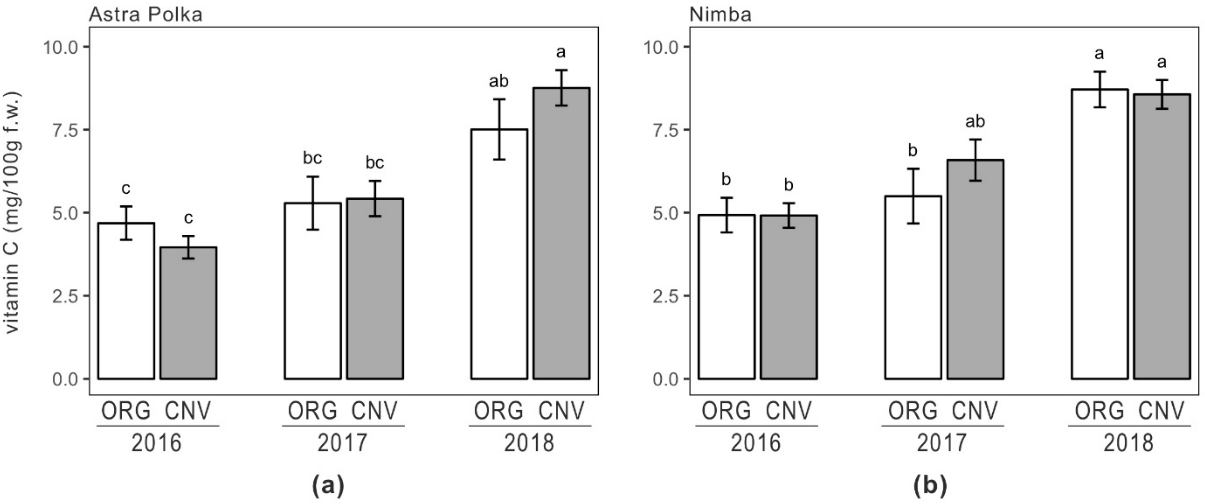

Figure S4. Vitamin C content in organic (ORG) and conventional (CNV) courgette fruits of (**a**) Astra Polka and (**b**) Nimba varieties in three years of cultivation. Data are presented as means with standard errors. Within each variety, bars marked with the same letters are not significantly different at the 5% level of probability.

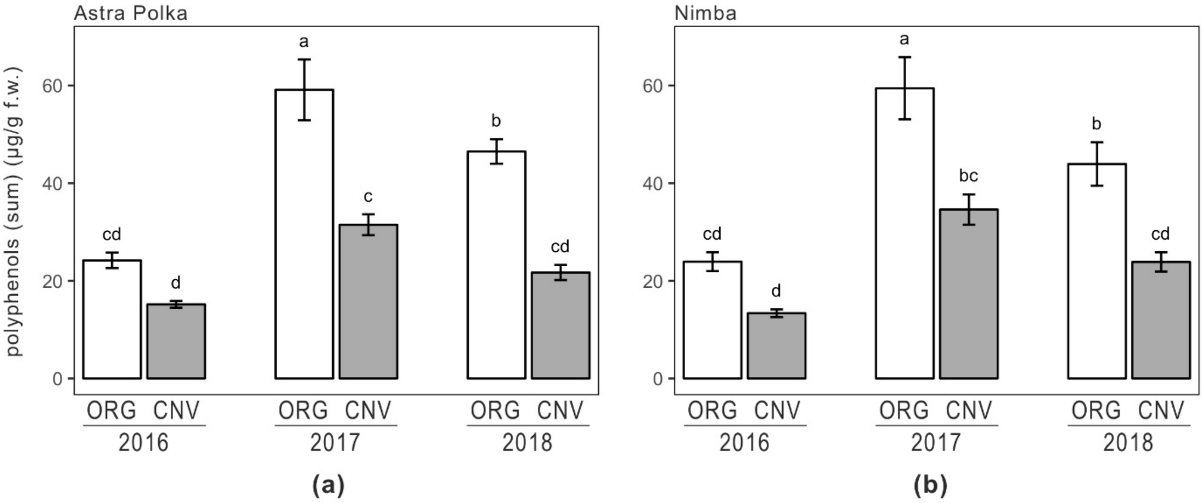

Figure S5. Polyphenols content in organic (ORG) and conventional (CNV) courgette fruits of (**a**) Astra Polka and (**b**) Nimba varieties in three years of cultivation. Data are presented as means with standard errors. Within each variety, bars marked with the same letters are not significantly different at the 5% level of probability.

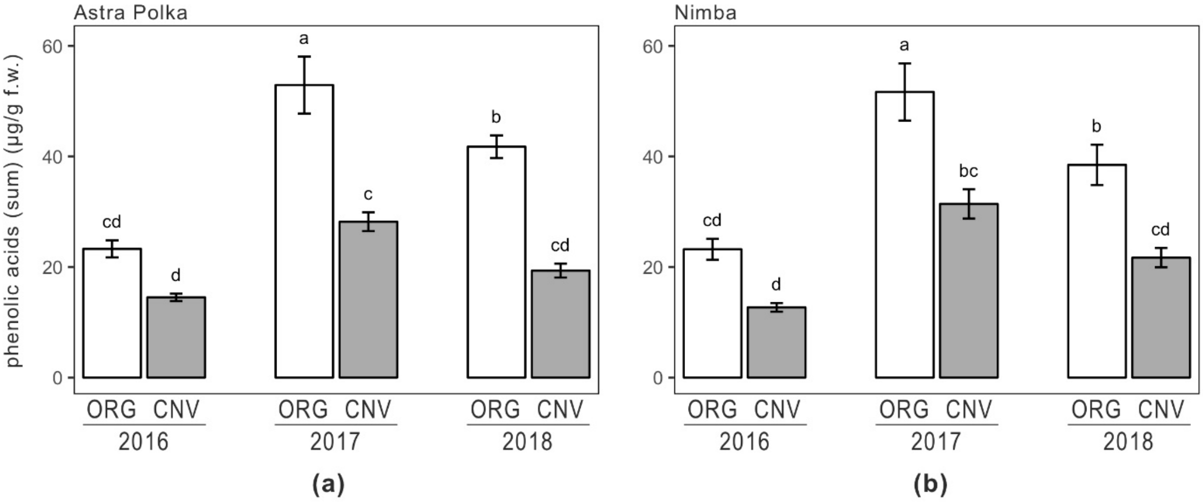

Figure S6. Phenolic acids (sum) content in organic (ORG) and conventional (CNV) courgette fruits of (**a**) Astra Polka and (**b**) Nimba varieties in three years of cultivation. Data are presented as means with standard errors. Within each variety, bars marked with the same letters are not significantly different at the 5% level of probability.

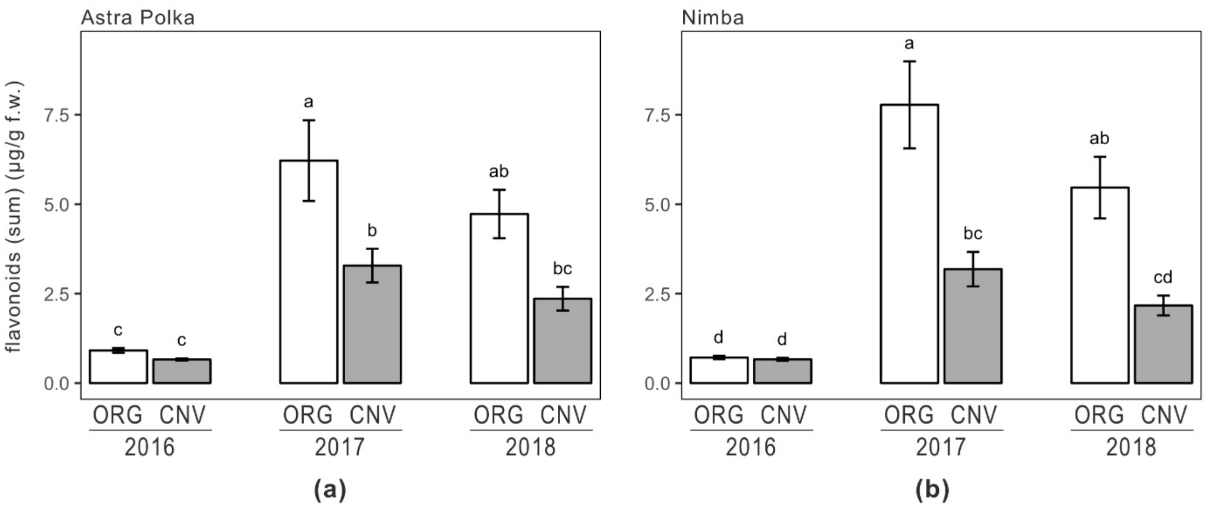

Figure S7. Flavonoids (sum) content in organic (ORG) and conventional (CNV) courgette fruits of (**a**) Astra Polka and (**b**) Nimba varieties in three years of cultivation. Data are presented as means with standard errors. Within each variety, bars marked with the same letters are not significantly different at the 5% level of probability.

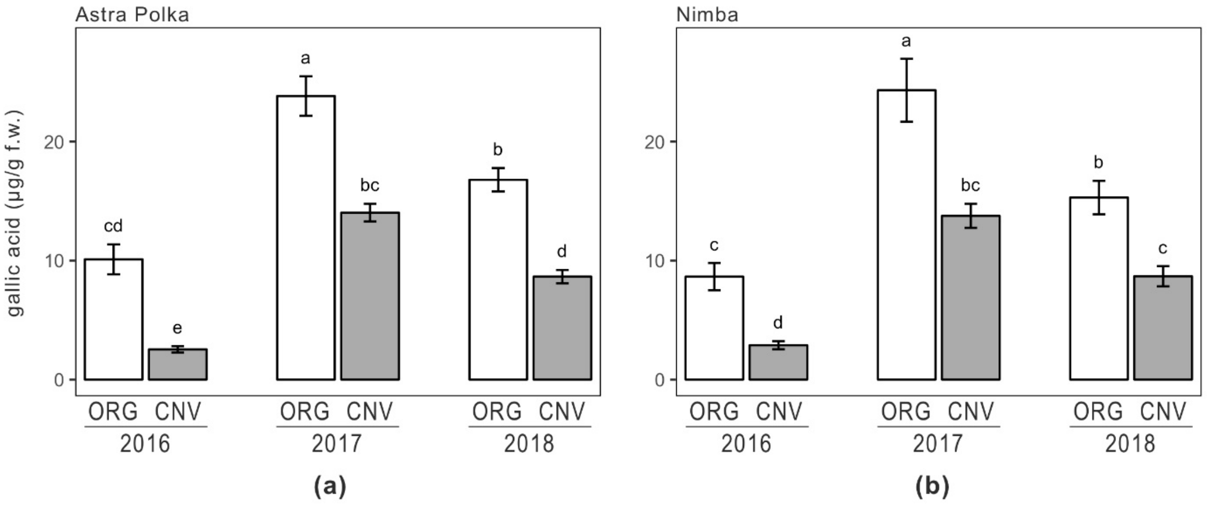

Figure S8. Gallic acid content in organic (ORG) and conventional (CNV) courgette fruits of (**a**) Astra Polka and (**b**) Nimba varieties in three years of cultivation. Data are presented as means with standard errors. Within each variety, bars marked with the same letters are not significantly different at the 5% level of probability.

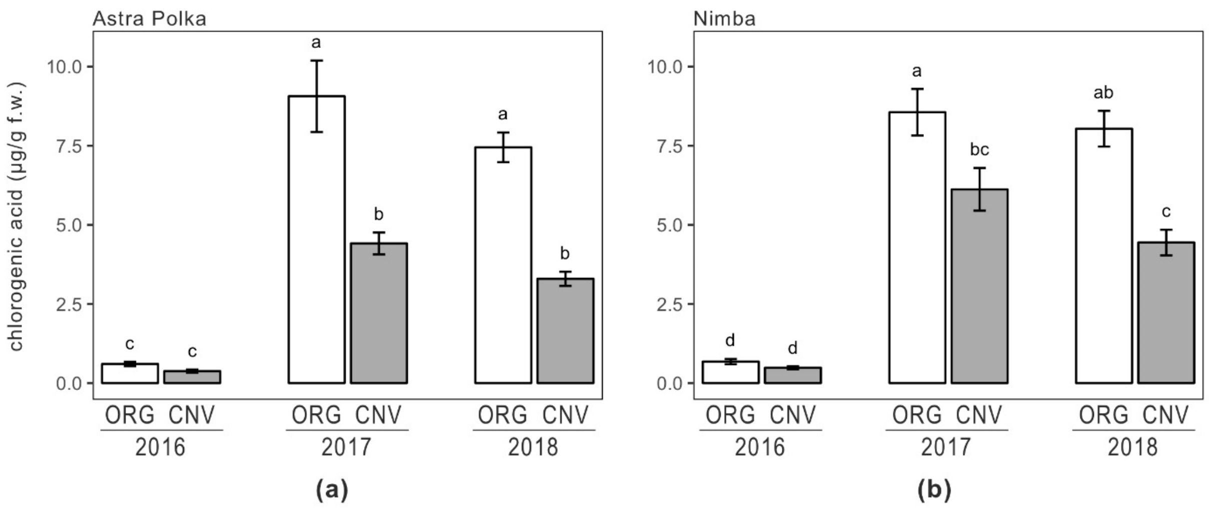

Figure S9. Chlorogenic acid content in organic (ORG) and conventional (CNV) courgette fruits of (**a**) Astra Polka and (**b**) Nimba varieties in three years of cultivation. Data are presented as means with standard errors. Within each variety, bars marked with the same letters are not significantly different at the 5% level of probability.

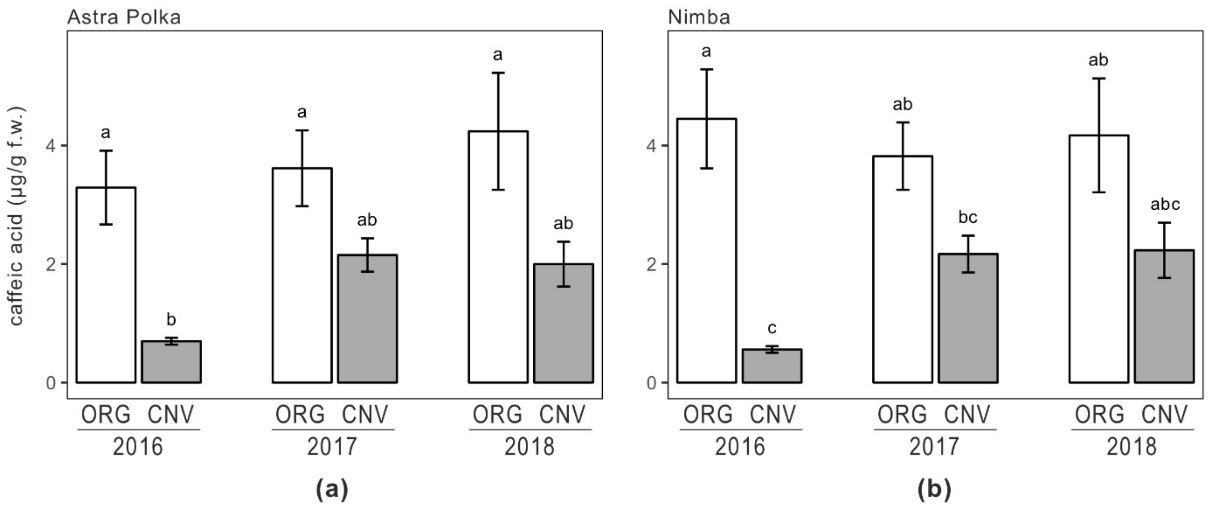

Figure S10. Caffeic acid content in organic (ORG) and conventional (CNV) courgette fruits of (**a**) Astra Polka and (**b**) Nimba varieties in three years of cultivation. Data are presented as means with standard errors. Within each variety, bars marked with the same letters are not significantly different at the 5% level of probability.

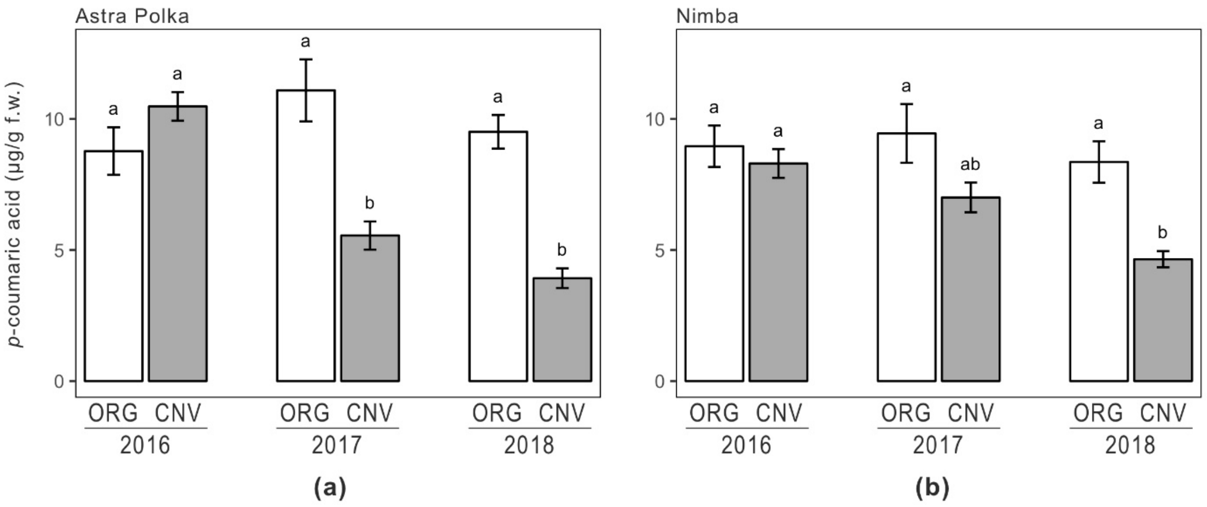

Figure S11. *p*-coumaric acid content in organic (ORG) and conventional (CNV) courgette fruits of (**a**) Astra Polka and (**b**) Nimba varieties in three years of cultivation. Data are presented as means with standard errors. Within each variety, bars marked with the same letters are not significantly different at the 5% level of probability.

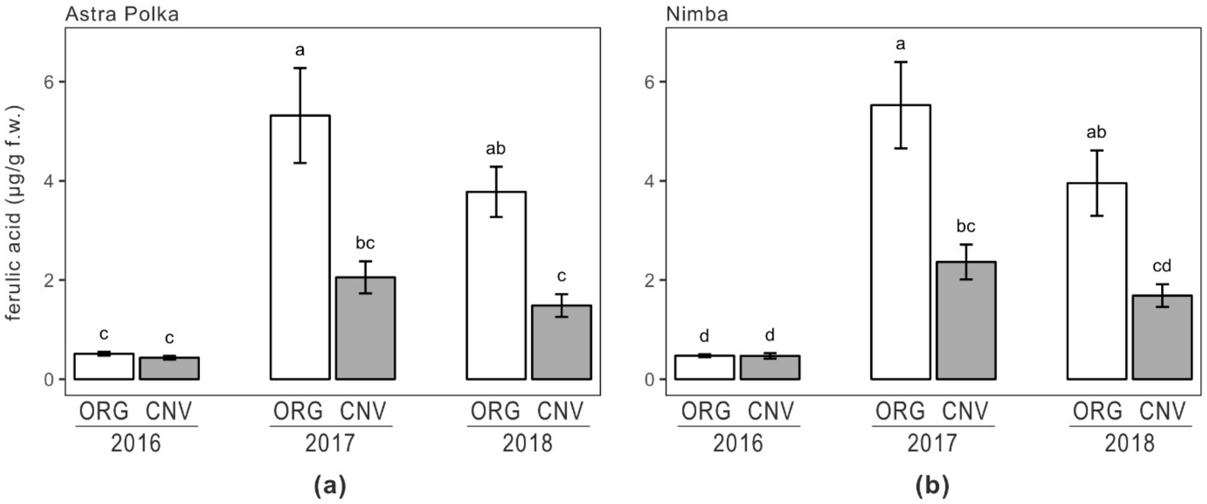

Figure S12. Ferulic acid content in organic (ORG) and conventional (CNV) courgette fruits of (**a**) Astra Polka and (**b**) Nimba varieties in three years of cultivation. Data are presented as means with standard errors. Within each variety, bars marked with the same letters are not significantly different at the 5% level of probability.

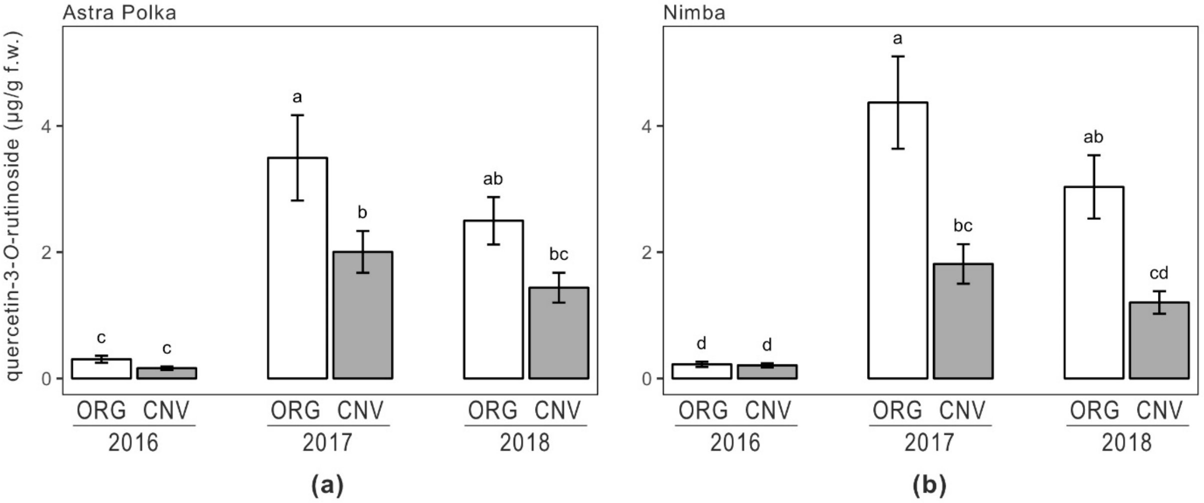

Figure S13. Quercetin-3-*O*-rutinoside content in organic (ORG) and conventional (CNV) courgette fruits of (**a**) Astra Polka and (**b**) Nimba varieties in three years of cultivation. Data are presented as means with standard errors. Within each variety, bars marked with the same letters are not significantly different at the 5% level of probability.

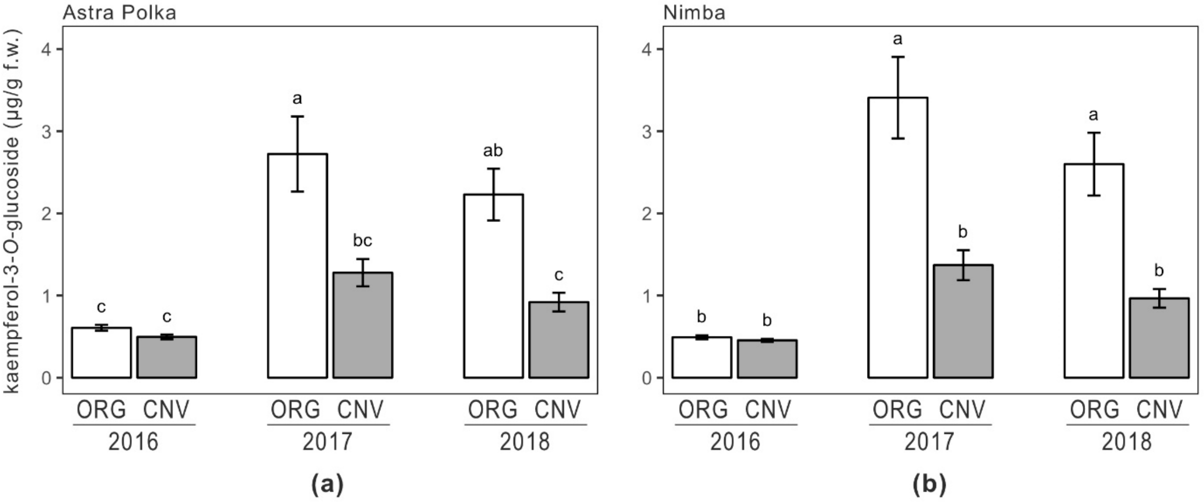

Figure S14. Kaempferol-3-*O*-glucoside content in organic (ORG) and conventional (CNV) courgette fruits of (**a**) Astra Polka and (**b**) Nimba varieties in three years of cultivation. Data are presented as means with standard errors. Within each variety, bars marked with the same letters are not significantly different at the 5% level of probability.

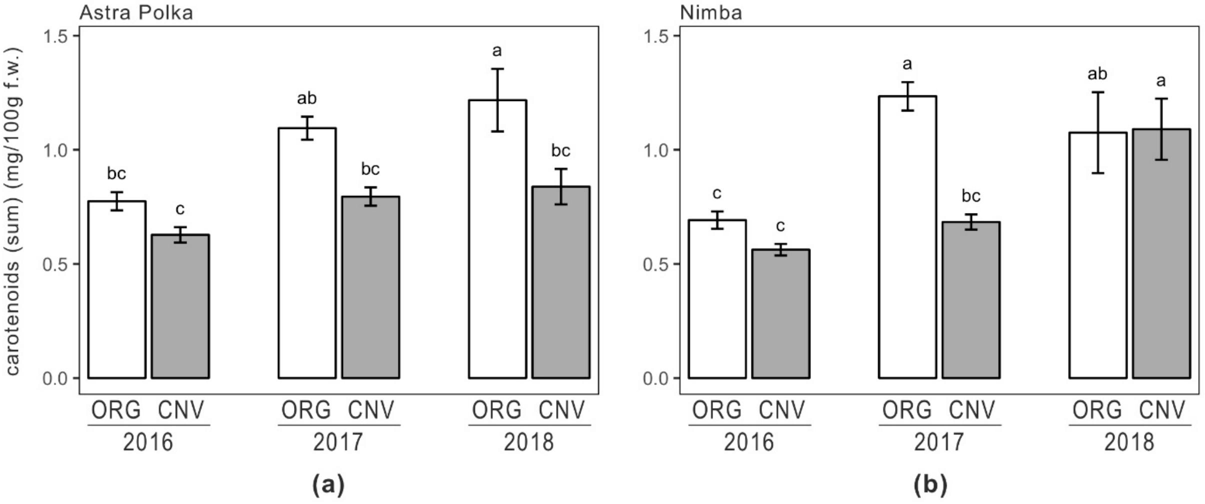

Figure S15. Carotenoids (sum) content in organic (ORG) and conventional (CNV) courgette fruits of (**a**) Astra Polka and (**b**) Nimba varieties in three years of cultivation. Data are presented as means with standard errors. Within each variety, bars marked with the same letters are not significantly different at the 5% level of probability.

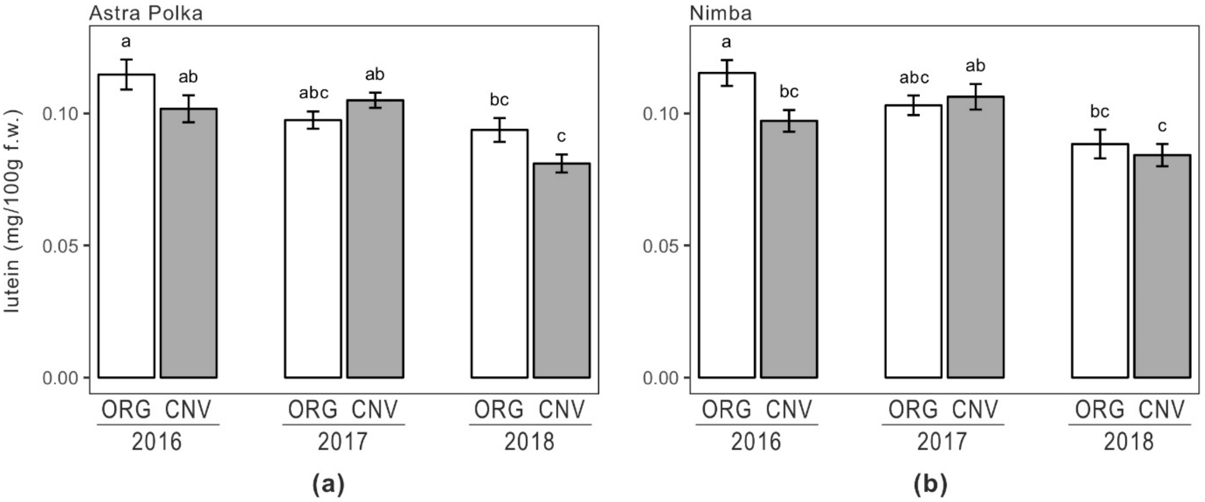

Figure S16. Lutein content in organic (ORG) and conventional (CNV) courgette fruits of (**a**) Astra Polka and (**b**) Nimba varieties in three years of cultivation. Data are presented as means with standard errors. Within each variety, bars marked with the same letters are not significantly different at the 5% level of probability.

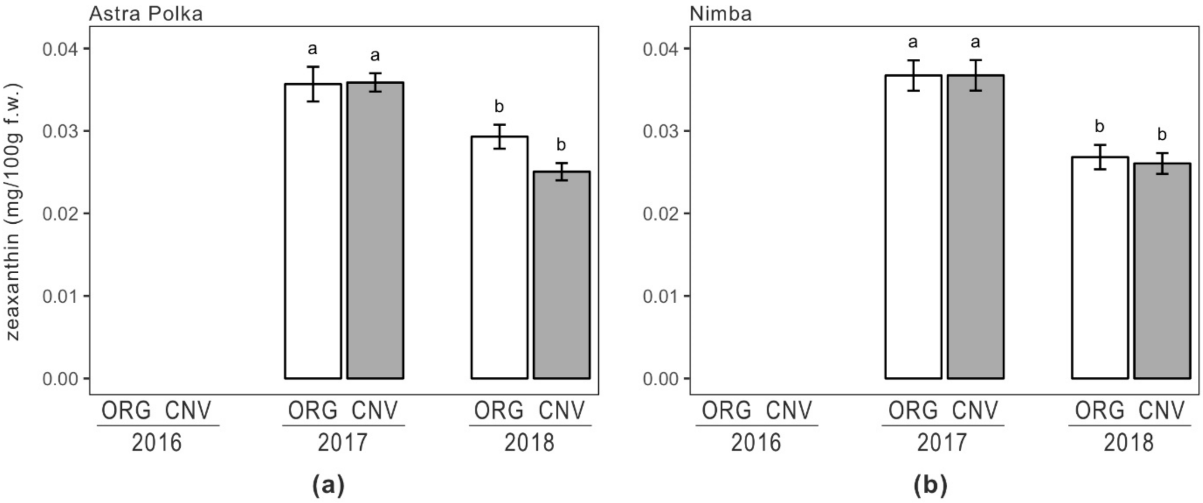

Figure S17. Zeaxanthin content in organic (ORG) and conventional (CNV) courgette fruits of (**a**) Astra Polka and (**b**) Nimba varieties in three years of cultivation. Data are presented as means with standard errors. Within each variety, bars marked with the same letters are not significantly different at the 5% level of probability.

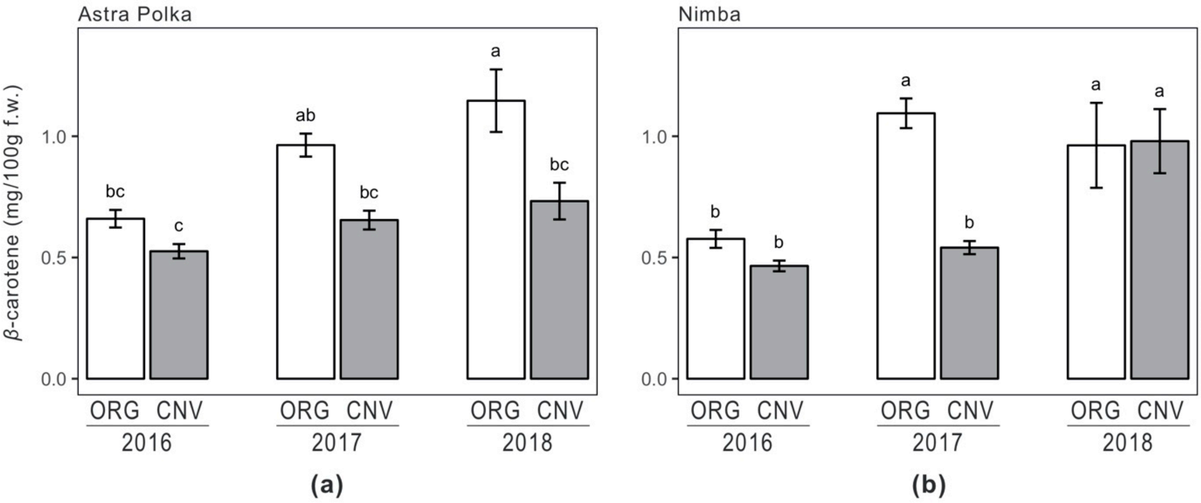

Figure S18. β-carotene content in organic (ORG) and conventional (CNV) courgette fruits of (**a**) Astra Polka and (**b**) Nimba varieties in three years of cultivation. Data are presented as means with standard errors. Within each variety, bars marked with the same letters are not significantly different at the 5% level of probability.

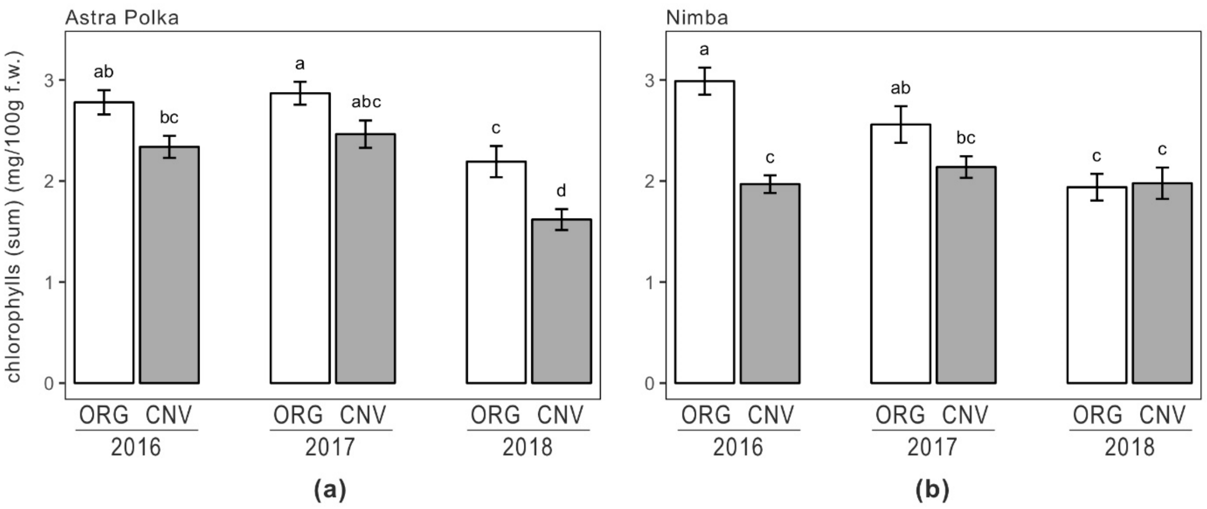

Figure S19. Chlorophylls (sum) content in organic (ORG) and conventional (CNV) courgette fruits of (**a**) Astra Polka and (**b**) Nimba varieties in three years of cultivation. Data are presented as means with standard errors. Within each variety, bars marked with the same letters are not significantly different at the 5% level of probability.

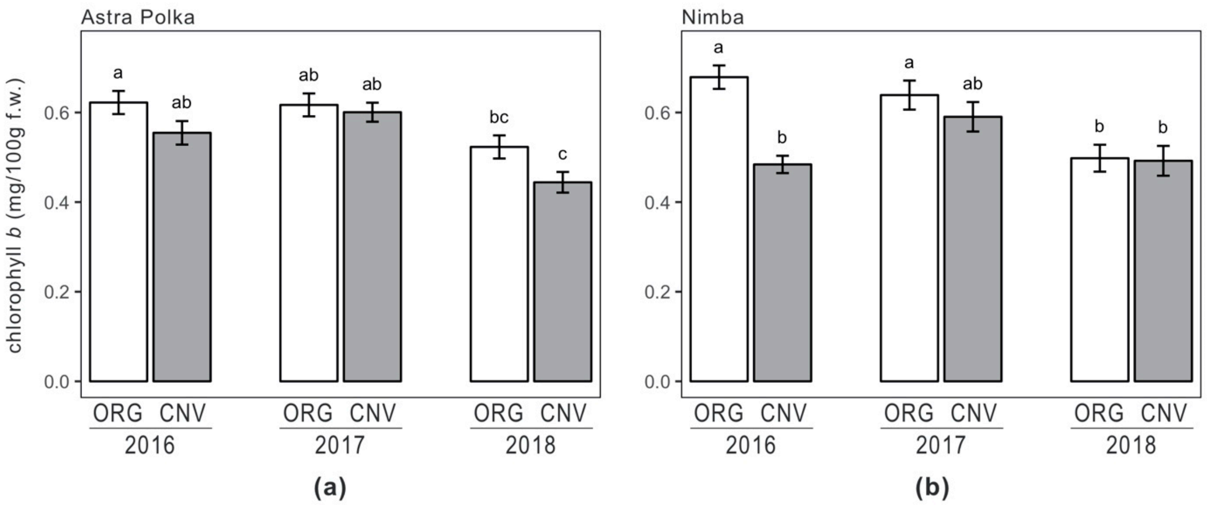

Figure S20. Chlorophyll *b* content in organic (ORG) and conventional (CNV) courgette fruits of (**a**) Astra Polka and (**b**) Nimba varieties in three years of cultivation. Data are presented as means with standard errors. Within each variety, bars marked with the same letters are not significantly different at the 5% level of probability.

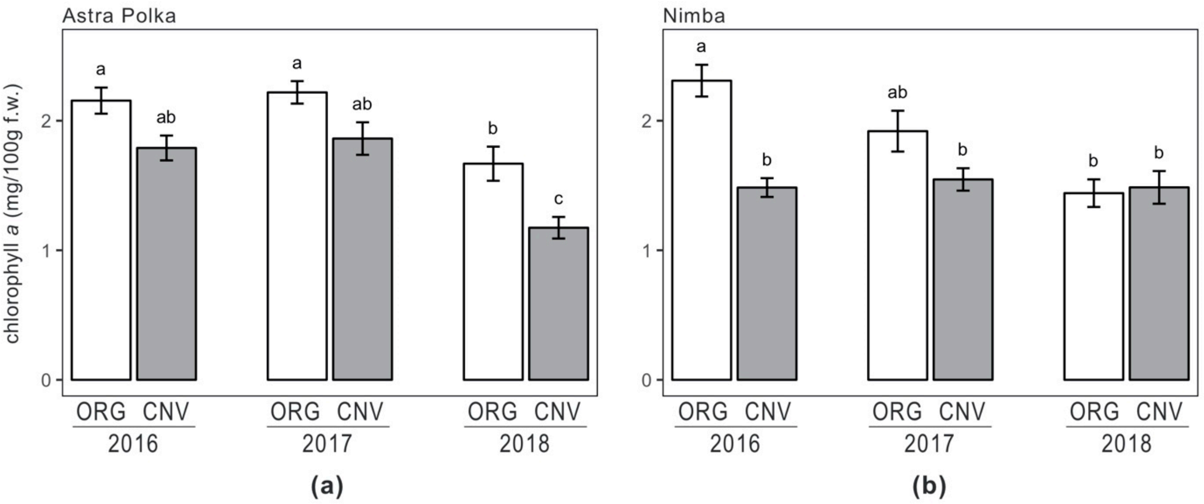

Figure S21. Chlorophyll *a* content in organic (ORG) and conventional (CNV) courgette fruits of (**a**) Astra Polka and (**b**) Nimba varieties in three years of cultivation. Data are presented as means with standard errors. Within each variety, bars marked with the same letters are not significantly different at the 5% level of probability.
